# Crystal structure and Hirshfeld surface analysis of bis­(benzoyl­acetonato)(ethanol)dioxidouranium(VI)

**DOI:** 10.1107/S2056989024010417

**Published:** 2024-11-05

**Authors:** Xolida Jabborova, Xusnida Tursinboyeva, Bakhtigul Ruzieva, Kambarali Turgunov, Jamshid Ashurov, Akmaljon Tojiboev, Shahlo Daminova

**Affiliations:** a Institute of General and Inorganic Chemistry, Academy of Sciences of Uzbekistan, 100170, M. Ulugbek Str 77a, Tashkent, Uzbekistan; b National University of Uzbekistan named after Mirzo Ulugbek University Street 4 Tashkent 100174 Uzbekistan; c Alfraganus University, 100190, Uzbekistan, Tashkent, Yunusabad district, Yukori Karakamish Street 2, Uzbekistan; d Uzbekistan–Japan Innovation Center of Youth, University Street 2B, Tashkent 100095, Uzbekistan; e S. Yunusov Institute of the Chemistry of Plant Substances Academy of Sciences of Uzbekistan Mirzo Ulugbek Str 77 Tashkent 100170 Uzbekistan; f Turin Polytechnic University in Tashkent Kichik Khalka Yuli Str 17 100095 Tashkent Uzbekistan; g Institute of Bioorganic Chemistry, Academy of Sciences of Uzbekistan, 100125, M. Ulugbek Str. 83, Tashkent, Uzbekistan; h University of Geological Sciences, Olimlar Street, 64, Mirzo Ulugbek district, Tashkent, Uzbekistan; i Namangan State University, Boburshoh str. 161, Namangan, 160107, Uzbekistan; Illinois State University, USA

**Keywords:** benzoyl acetone, uranium complex, crystal structure, Hirshfeld surface analysis, hydrogen bonding, π–π inter­action

## Abstract

A new uranium metal–organic complex salt, [U(C_10_H_9_O_2_)_2_O_2_(C_2_H_6_O)], with benzoyl acetone, namely, bis­(benzoyl­acetonato)(ethanol)dioxidouranium(VI), was synthesized. The compound has monoclinic *P*2_1_/*n* symmetry. The geometry of the seven-coordinate U atom is penta­gonal bipyramidal, with the uranyl oxygen atoms in apical positions. In the complex, the ligands bind to the metal through oxygen atoms. Additional weak O—H⋯O contacts between the cations and anions consolidate the three-dimensional arrangement of the structure. On the Hirshfeld surface, the largest contributions come from the short contacts such as van der Waals forces, including H⋯H, O⋯H and C⋯H. Inter­actions including C⋯C and O⋯C contacts were also observed; however, their contribution to the overall cohesion of the crystal structure is minor. A packing analysis was performed to check the strength of the crystal packing.

## Abstract 2

In the complex, the ligand binds to the metal through an oxygen atom. The geometry of the seven-coordinate U atom is penta­gonal bipyramidal, with the uranyl O atoms in apical positions.

## 1. Chemical context

A greater understanding of the coordination chemistry of uranium is important for the development of new technologies for the safe reprocessing and long-term immobilization of irradiated nuclear fuel. One of the main reasons for the renewed inter­est in uranium compounds is their remarkable structural versatility. In the oxidation states +III or +IV, eight- or nine-coordinate uranium environments are typically found, similar to those observed in lanthanide complexes (Enriquez *et al.*, 2005 ▸; Oldham *et al.* 2002 ▸). Uranium oxo compounds with oxidation state +VI form approximately linear triatomic uranyl ions, UO_2_^2+^. Although this cation can bind additional ligands perpendicular to the uranium axis to form five-, six-, seven-, and eight-coordinate metal centres, seven-coordinate is particularly common for hexa­valent uranium oxo compounds (Hernandez *et al.*, 2022 ▸; Almond & Albrecht-Schmitt, 2003 ▸; Arndt *et al.* 2002 ▸). Heptacoordinated uranium centers can exhibit penta­gonal–bipyramidal, capped-octa­hedral, and trigonal–prismatic coordination geometries. The specific geometry depends on steric requirements caused by ligand–ligand repulsion, weaker bonds, and generally reduced crystal field stability. Despite the abundance of layered structures for U^VI^–oxo compounds (Chakraborty *et al.*, 2006 ▸; Hughes & Burns, 2003 ▸; Neu *et al.*, 2001 ▸), one-dimensional topology or multidimensional framework structural studies of uranyl compounds are rather sparse (Bean *et al.*, 2001 ▸; Sykora & Albrecht-Schmitt, 2003 ▸). The anti-inflammatory, analgesic, anti-microbial, anti-convulsant, anti-cancer, anti-tubercular, anti­oxidant, anti­depressant, anti­glycation, anti­helmintic, anti-fungal, anti-tumour, anti­biotic and anti-allergic effects of the ligand have been studied (Şahin & Dege, 2021 ▸).
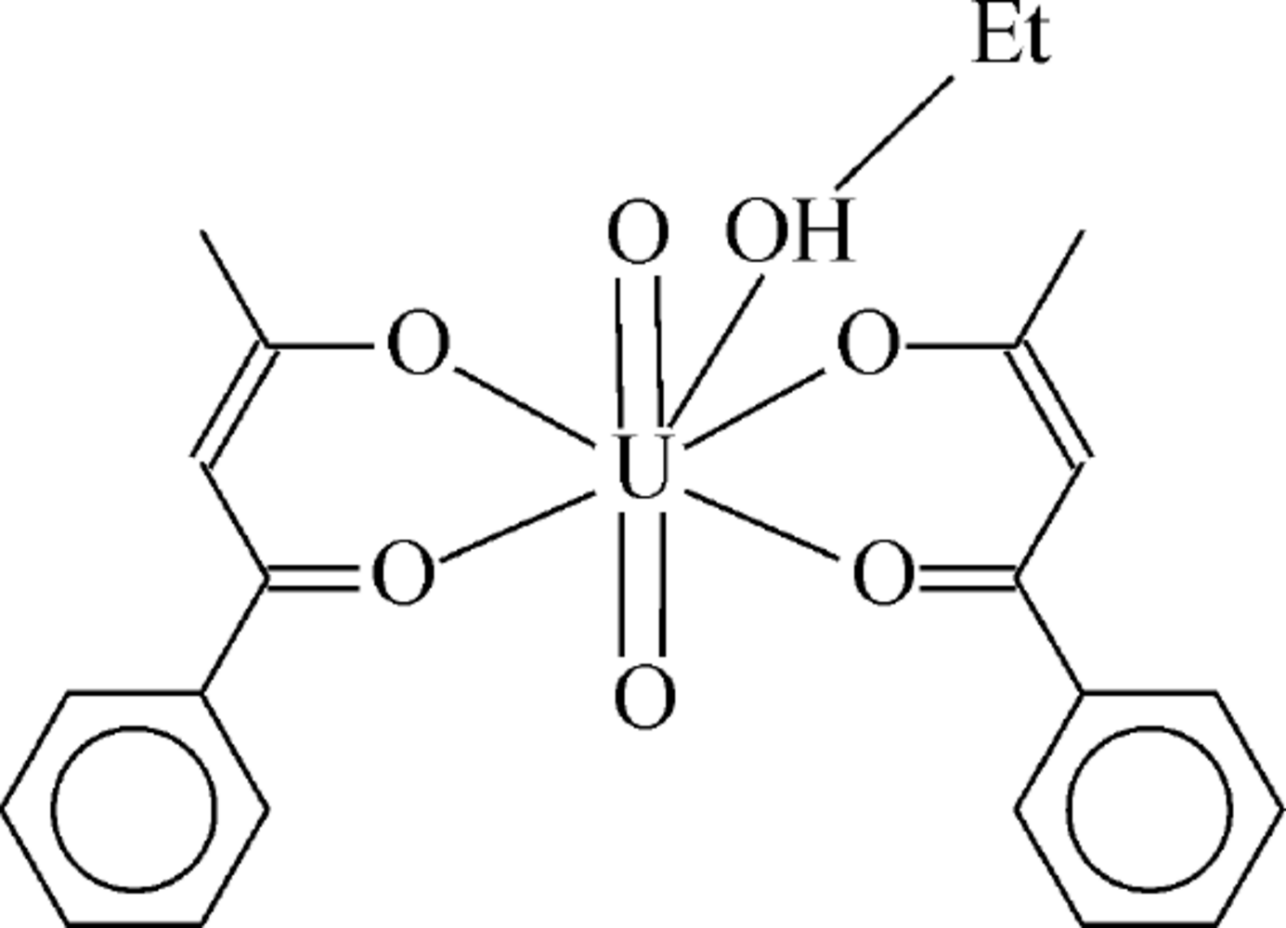


The present work was undertaken to study the effect of oxo-ligands on the metal coordination geometry and explore the possibility of any supra­molecular architecture in the resulting uranyl compounds. We isolated the title metal–organic complex uranium salt, [C_22_H_24_O_7_U] and report here its crystal structure and Hirshfeld surface analysis.

## 2. Structural commentary

**Figure 1:**
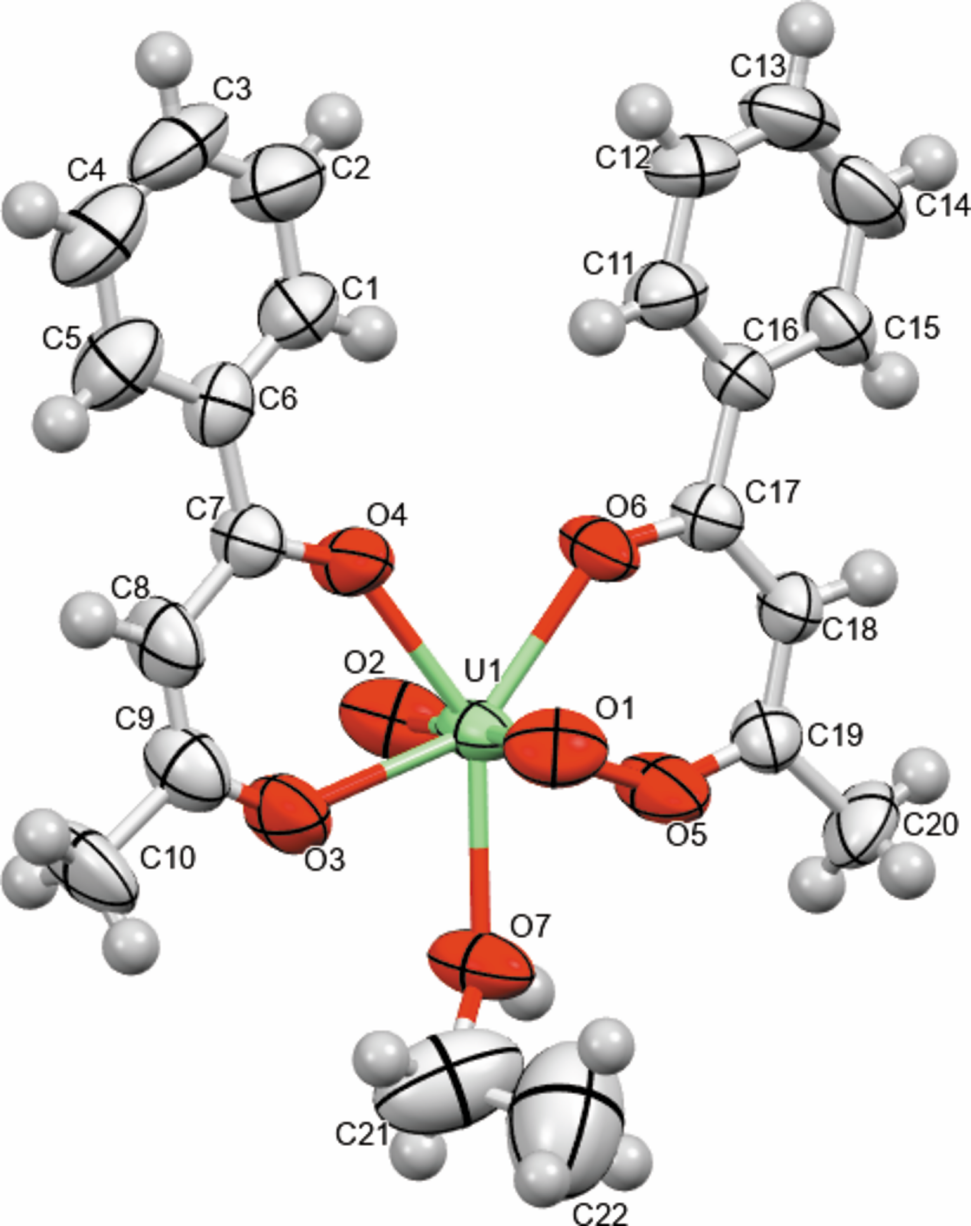
The mol­ecular structure of title compound with the atom-numbering scheme. Displacement ellipsoids are drawn at the 50% probability level and H atoms are displayed as small spheres of arbitrary radii.

**Figure 2:**
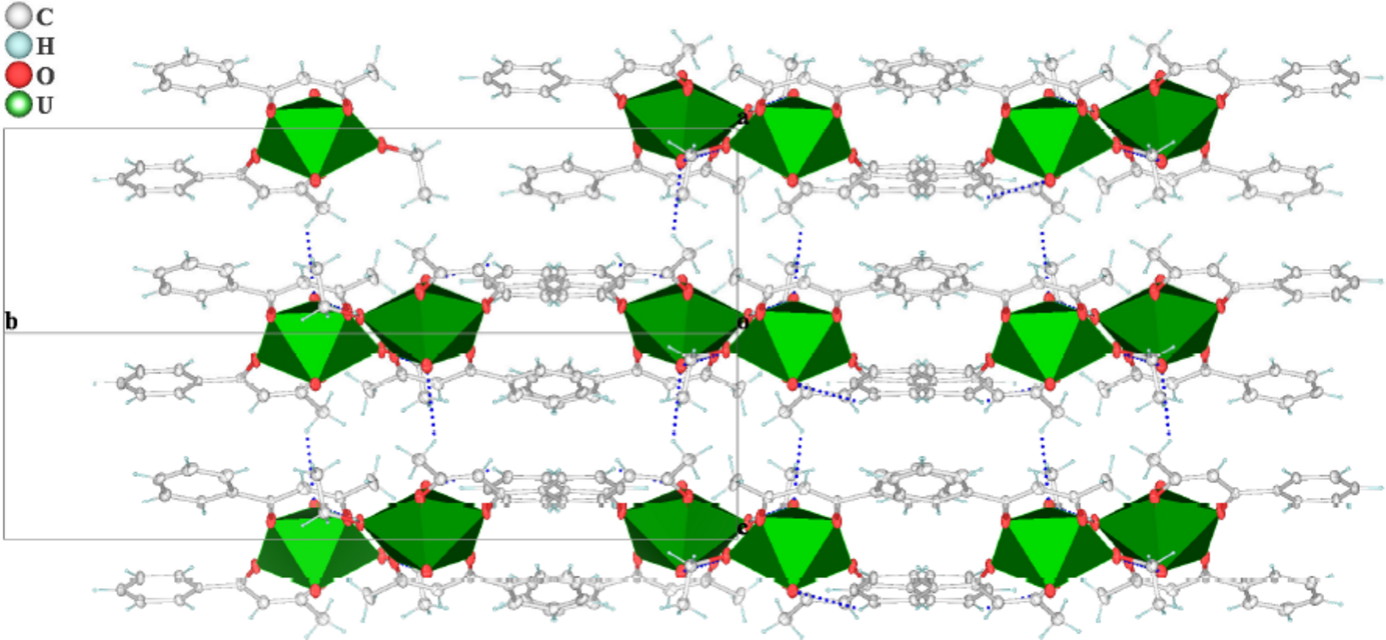
A view of the mol­ecular packing showing the penta­gonal–bipyramidal structure extending along the *b*-axis direction.

The single-crystal structure of bis­(benzoyl­acetonato)(ethanol)dioxidouranium(VI) crystallizes in the monoclinic space group *P*2_1_/*n*. The mol­ecular structure is shown in Fig. 1 ▸. The mol­ecule is almost planar with an r.m.s. deviation of 0.0593 Å from planarity. In the compound, the coordination geometry around the uranium atom includes seven oxygen donors from one ethanol, two oxido, and two bidentate benzoyl­acetonoate ligands. It is approximately penta­gonal bipyramidal. The U—O uranyl bond distances [1.759 (7) and 2.358 (7) Å; Table 1 ▸] agree well with the previously reported values for dioxouranium (VI) complexes (Hernandez *et al.*, 2022 ▸; Takao & Ikeda, 2008 ▸; Chakraborty *et al.*, 2006 ▸; Gatto *et al.*, 2004 ▸; Kannan *et al.*, 2004 ▸). The distortion of the metal coordination geometry from an ideal penta­gonal bipyramidal arrangement (Fig. 2 ▸) is revealed by the O—U—O bond angles for the penta­gon, which range between 70.6 (2) and 177.4 (3)°.

## 3. Supra­molecular features

**Figure 3:**
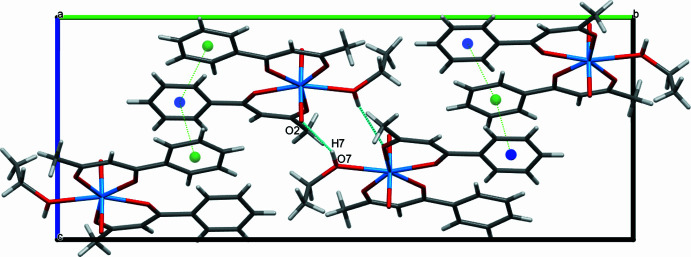
View of the crystal structure of the title compound, showing the O—H⋯O hydrogen bond and π–π inter­actions as green dotted lines.

In the complex, the crystal packing exhibits one inter­molecular O7—H7⋯O2(1 − *x*, 1 − *y*, 1 − *z*) hydrogen-bonding inter­actions (Fig. 3 ▸, Table 2 ▸). In additional π–π stacking (Fig. 3 ▸) occurs between the aromatic rings of neighbouring mol­ecules with centroid–centroid distances *Cg*1⋯*Cg*2(−1 + *x*, *y*, *z*) = 3.900 (6) Å, with a ring slippage of 1.577 Å, and *Cg*2⋯*Cg*1(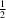 + *x*, 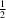 − *y*, 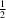 + *z*) = 3.765 (6) Å, with a ring slippage of 1.035 Å where *Cg*1 and *Cg*2 are the centroids of the C1–C6 and C11–C16 rings, respectively.

## 4. Hirshfeld surface analysis

**Figure 4:**
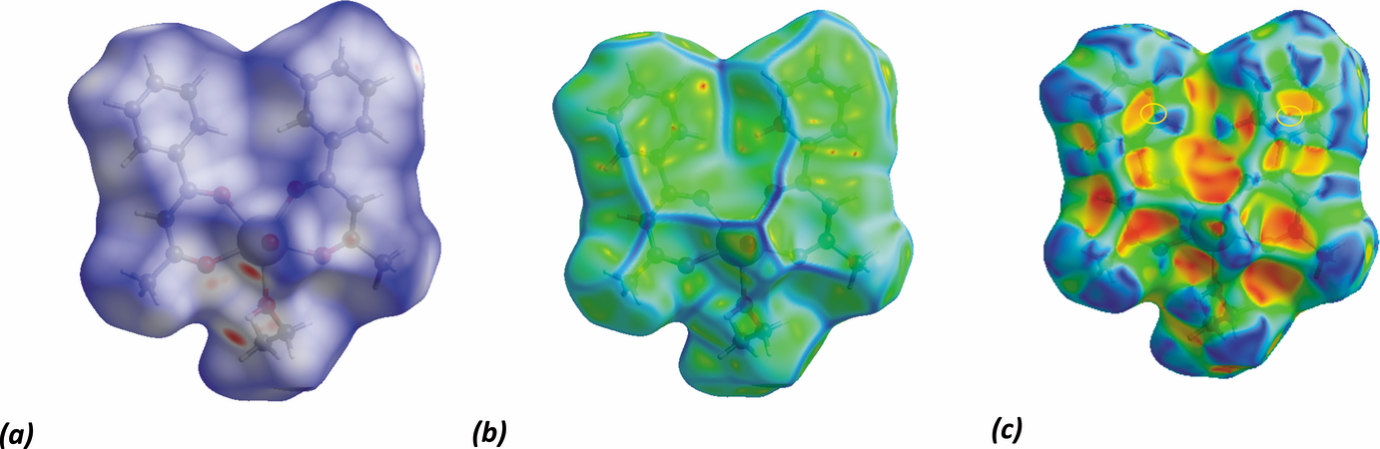
Hirshfeld surfaces of the title complex mapped with (*a*) *d*_norm_, (*b*) curvedness and (*c*) shape-index.

To further investigate the inter­molecular inter­actions present in the title compound, a Hirshfeld surface (HS) analysis was performed, and the two-dimensional fingerprint plots were generated with *CrystalExplorer17.5* (Spackman *et al.*, 2021 ▸). The HS mapped with *d*_norm_, curvedness and shape-index are given in Fig. 4 ▸. The white surface indicates contacts with distances equal to the sum of van der Waals radii, and the red and blue colours indicate distances shorter or longer than the van der Waals radii, respectively. The bright-red points in the *d*_norm_ surface of the mol­ecule are located near atoms O2 and O7/H7, consistent with the O7—H7⋯O2 hydrogen-bonding inter­action, highlighted in Fig. 3 ▸. From the Hirshfeld surfaces, it is also evident that the mol­ecules are related to one another by π–π stacking inter­actions, as can be inferred from inspection of the adjacent red and blue triangles (highlighted by yellow circles) on the shape-index surface (Fig. 4 ▸). The presence of π–π stacking is also evident in the flat region toward the top of both sides of the mol­ecules and is clearly visible on the curvedness surface (Fig. 4 ▸): the shape of the blue outline on the curvedness surface unambiguously delineates the contacting patches of the mol­ecules.

**Figure 5:**
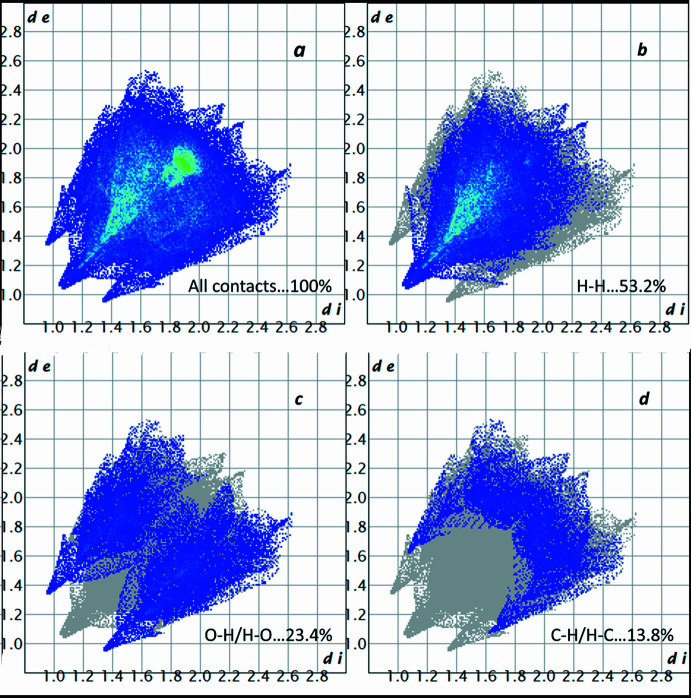
Two-dimensional fingerprint plots for the title compound, showing (*a*) all inter­actions, and decomposed into (*b*) H⋯H, (*c*) O⋯H/H⋯O and (*d*) C⋯H/H⋯C inter­actions.

The two-dimensional (2D) fingerprint plots (McKinnon *et al.*, 2007 ▸) are shown in Fig. 5 ▸. On the HS, the largest contributions (53.2%, 23.4%, 13.8%) come from short contacts such as van der Waals forces, H⋯H, O⋯H and C⋯H contacts. C⋯C (8.6%), O⋯C (0.8%) and O⋯O (0.1%) contacts are also observed. The classical O—H⋯O hydrogen bonds correspond to O⋯H/H⋯O contacts (23.4% contribution) in Fig. 5 ▸ and show up as a pair of spikes. The scattered points in the breakdown of the fingerprint plot show that the π–π stacking inter­actions C⋯C comprise 8.6% of the total Hirshfeld surface of the mol­ecule displayed as a region of blue/green colour.

## 5. Database survey

A search in the Cambridge Structural Database (CSD, version 5.43, update of November 2022; Groom *et al.*, 2016 ▸) revealed 25 hits with the *β*-diketonate ligand moiety. Among these, one structure contains tin (AGESUA: Pettinari *et al.*, 2002 ▸), two structures contain zinc (BZACZN: Belford *et al.*, 1969 ▸; NEYBID: Dang *et al.*, 2006 ▸), one structure contains uranium(VI) (CAZMEV: Haider *et al.*, 1983 ▸), one structure contains platinum(II) (CBZACP: Okeya *et al.*, 1976 ▸), one structure contains iron(III) (ARUMOR: Zou *et al.*, 2016 ▸), one structure contains manganese(II), one structure contains cadmium (HICRAP and HICRET: Yang, 2018*a* ▸,*b* ▸), one structure contains vanadium (KIJPAV: Xing *et al.*, 2007 ▸), four structures contain copper (CUBEAC: Hon *et al.*, 1966 ▸; LEZVAO: Lennartson *et al.*, 2007 ▸; NINFIC, NINFOI: Chen *et al.*, 2018 ▸), one structure contains lithium (UCIMAU: Jung *et al.*, 1998 ▸), nine structures contain manganese(II) (NENNAX: Cvrtila *et al.*, 2012 ▸; PIDPOJ, PIDPUP, PIDQAW, PIDQEA, PIDQIE, PIDQOK, PIDQUQ, PIDRAX: Cvrtila *et al.*, 2013 ▸), and two structures contain cobalt(II) (POJBUN: Perdih, 2014 ▸; YADKUJ: Döring *et al.*, 1992 ▸). A search for the uranyl moiety returned five hits with penta­gonal–bipyramidal geometries similar to that in the title structure. These include: aqua­bis­(benzoyl­acetonato)dioxouranium(VI) monohydrate (CAZMEV: Haider *et al.*, 1983 ▸), uran­yl(VI) complexes containing the *β*-diketonatephenol ligands derived from 1-(2-hy­droxy­phen­yl)-1,3-butane­dione and 1-(2-hy­droxy­phen­yl)-3-phenyl-1,3-propane­dione (GIYXAN, GIYXER: Ainscough *et al.*, 1998 ▸), a uranyl *β*-diketonate complex [UO_2_(tfa)_2_(*L*)] [*L* = H_2_O, OHCH_2_CH_3_; tfa = deprotonated 4,4,4,-tri­fluoro-1-(2-fur­yl)-1,3-butane­dione] with a well-described 3D supra­molecular structure and electronic absorption spectroscopy (IVEDIX: Al-Anber *et al.*, 2011 ▸), and bis­(2-benzoyl-1-phenyl­ethenolato-*κ*^2^*O*,*O′*)(ethanol-*κO*)dioxidouranium(VI) (RISVAR: Takao & Ikeda, 2008 ▸).

## 6. Synthesis and crystallization

Benzoyl­acetone (BNA) (0.0324 g, 0.200 mmol) dissolved in 5 ml of ethanol and uranyl acetate (0.0388 g, 0.100 mmol) dissolved in 5 ml of ethanol were mixed under constant stirring until the colour of the solution turned to orange–red. The stirring continued for an hour, then the solution was left to stand overnight. The orange–red crystalline solid was filtered off and dried under vacuum. The solid was dissolved in ethanol and slow evaporation of the solution yielded diffraction-quality single-crystals of the title compound. Selected IR bands (KBr pellet, cm^−1^): 1589 (C=O), 1340 (C—O), 471 (U—O_ligand_), 380 (U—O_eth_ Raman spectroscopy), 908 (O=U=O).

## 7. Refinement

Crystal data, data collection and structure refinement details are summarized in Table 3 ▸. C-bound H atoms were positioned geometrically (C—H = 0.93–0.97 Å) and treated as riding on their parent atoms, with C—H = 0.95 Å (aromatic) and were refined with *U*_iso_(H) = 1.2*U*_eq_(C).

## Supplementary Material

Crystal structure: contains datablock(s) I. DOI: 10.1107/S2056989024010417/ej2006sup1.cif

Structure factors: contains datablock(s) I. DOI: 10.1107/S2056989024010417/ej2006Isup2.hkl

CCDC reference: 2291369

Additional supporting information:  crystallographic information; 3D view; checkCIF report

## Figures and Tables

**Table 1:** Selected bond lengths (Å)

U1—O6	2.317 (5)	U1—O1	1.759 (7)
U1—O7	2.458 (5)	U1—O3	2.358 (7)
U1—O4	2.308 (5)	U1—O5	2.369 (7)
U1—O2	1.779 (8)		

**Table 2:** Hydrogen-bond geometry (Å, °)

*D*—H⋯*A*	*D*—H	H⋯*A*	*D*⋯*A*	*D*—H⋯*A*
O7—H7⋯O2^i^	0.86 (6)	2.44 (5)	3.246 (10)	158 (3)

Symmetry code: (i) 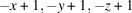.

**Table 3:** Experimental details

Crystal data	
Chemical formula	[U(C_10_H_9_O_2_)_2_O_2_(C_2_H_6_O)]
*M*_r_	638.44
Crystal system, space group	Monoclinic, *P*2_1_/*n*
Temperature (K)	293
*a*, *b*, *c* (Å)	8.55214 (16), 26.0026 (4), 10.3057 (2)
β (°)	102.8291 (19)
*V* (Å^3^)	2234.56 (7)
*Z*	4
Radiation type	Cu *K*α
μ (mm^−1^)	20.79
Crystal size (mm)	0.28 × 0.26 × 0.18

Data collection	
Diffractometer	XtaLAB Synergy, Single source at home/near, HyPix3000
Absorption correction	Multi-scan (*CrysAlis PRO*; Rigaku OD, 2020 ▸)
*T*_min_, *T*_max_	0.266, 1.000
No. of measured, independent and observed [*I* > 2σ(*I*)] reflections	22840, 4331, 3260
*R*_int_	0.085
(sin θ/λ)_max_ (Å^−1^)	0.615

Refinement	
*R*[*F*^2^ > 2σ(*F*^2^)], *wR*(*F*^2^), *S*	0.041, 0.115, 1.05
No. of reflections	4331
No. of parameters	277
No. of restraints	4
H-atom treatment	H atoms treated by a mixture of independent and constrained refinement
Δρ_max_, Δρ_min_ (e Å^−3^)	1.49, −1.95

Computer programs: *CrysAlis PRO* (Rigaku OD, 2020 ▸), *SHELXT2018/2* (Sheldrick, 2015*a* ▸), *SHELXL2019/3* (Sheldrick, 2015*b* ▸), *PLATON* (Spek, 2020 ▸) and *publCIF* (Westrip, 2010 ▸).

## supplementary crystallographic information

### Bis(benzoylacetonato)(ethanol)dioxidouranium(VI) Crystal data

**Table:**

[U(C_10_H_9_O_2_)_2_O_2_(C_2_H_6_O)]	*F*(000) = 1216
*M**_r_* = 638.44	*D*_x_ = 1.898 Mg m^−^^3^
Monoclinic, *P*2_1_/*n*	Cu *K*α radiation, λ = 1.54184 Å
*a* = 8.55214 (16) Å	Cell parameters from 7001 reflections
*b* = 26.0026 (4) Å	θ = 3.4–70.9°
*c* = 10.3057 (2) Å	µ = 20.79 mm^−^^1^
β = 102.8291 (19)°	*T* = 293 K
*V* = 2234.56 (7) Å^3^	Block, orange
*Z* = 4	0.28 × 0.26 × 0.18 mm

### Bis(benzoylacetonato)(ethanol)dioxidouranium(VI) Data collection

**Table:**

XtaLAB Synergy, Single source at home/near, HyPix3000 diffractometer	4331 independent reflections
Radiation source: micro-focus sealed X-ray tube, PhotonJet (Cu) X-ray Source	3260 reflections with *I* > 2σ(*I*)
Mirror monochromator	*R*_int_ = 0.085
Detector resolution: 10.0000 pixels mm^-1^	θ_max_ = 71.6°, θ_min_ = 3.4°
ω scans	*h* = −10→10
Absorption correction: multi-scan (CrysAlisPro; Rigaku OD, 2020)	*k* = −31→31
*T*_min_ = 0.266, *T*_max_ = 1.000	*l* = −12→12
22840 measured reflections	

### Bis(benzoylacetonato)(ethanol)dioxidouranium(VI) Refinement

**Table:**

Refinement on *F*^2^	4 restraints
Least-squares matrix: full	Hydrogen site location: mixed
*R*[*F*^2^ > 2σ(*F*^2^)] = 0.041	H atoms treated by a mixture of independent and constrained refinement
*wR*(*F*^2^) = 0.115	*w* = 1/[σ^2^(*F*_o_^2^) + (0.0559*P*)^2^] where *P* = (*F*_o_^2^ + 2*F*_c_^2^)/3
*S* = 1.05	(Δ/σ)_max_ = 0.001
4331 reflections	Δρ_max_ = 1.49 e Å^−^^3^
277 parameters	Δρ_min_ = −1.94 e Å^−^^3^

### Bis(benzoylacetonato)(ethanol)dioxidouranium(VI) Special details

**Table:**

Geometry. All esds (except the esd in the dihedral angle between two l.s. planes) are estimated using the full covariance matrix. The cell esds are taken into account individually in the estimation of esds in distances, angles and torsion angles; correlations between esds in cell parameters are only used when they are defined by crystal symmetry. An approximate (isotropic) treatment of cell esds is used for estimating esds involving l.s. planes.

### Bis(benzoylacetonato)(ethanol)dioxidouranium(VI) Fractional atomic coordinates and isotropic or equivalent isotropic displacement parameters (Å^2^)

**Table:**

	*x*	*y*	*z*	*U*_iso_*/*U*_eq_	
U1	0.35139 (3)	0.42260 (2)	0.30596 (3)	0.06079 (13)	
O6	0.4734 (6)	0.3427 (2)	0.3413 (7)	0.0814 (19)	
O7	0.4126 (8)	0.5149 (2)	0.3315 (7)	0.089 (2)	
H7	0.492 (7)	0.5227 (10)	0.395 (6)	0.133*	
O4	0.1461 (6)	0.3644 (2)	0.2364 (7)	0.0834 (18)	
O2	0.3043 (11)	0.4237 (2)	0.4656 (7)	0.097 (2)	
O1	0.4002 (10)	0.4245 (2)	0.1490 (7)	0.086 (2)	
O3	0.1132 (8)	0.4682 (3)	0.2177 (7)	0.094 (2)	
O5	0.6224 (8)	0.4331 (2)	0.4195 (8)	0.109 (3)	
C17	0.6112 (8)	0.3228 (3)	0.3875 (7)	0.0546 (17)	
C16	0.6196 (9)	0.2656 (3)	0.3843 (7)	0.0553 (17)	
C19	0.7469 (10)	0.4058 (3)	0.4512 (8)	0.0612 (19)	
C18	0.7443 (8)	0.3533 (3)	0.4370 (8)	0.065 (2)	
H18	0.842014	0.336419	0.463399	0.077*	
C6	−0.0712 (9)	0.3112 (3)	0.1567 (8)	0.065 (2)	
C7	0.0003 (9)	0.3631 (3)	0.1833 (8)	0.063 (2)	
C11	0.4925 (11)	0.2381 (3)	0.3120 (9)	0.073 (2)	
H11	0.402282	0.255445	0.265396	0.088*	
C9	−0.0310 (12)	0.4571 (4)	0.1714 (10)	0.083 (3)	
C20	0.8965 (10)	0.4343 (4)	0.5093 (11)	0.088 (3)	
H20A	0.939075	0.449118	0.439123	0.132*	
H20B	0.973720	0.411031	0.560045	0.132*	
H20C	0.873495	0.461083	0.566368	0.132*	
C15	0.7523 (10)	0.2390 (3)	0.4547 (9)	0.074 (2)	
H15	0.840231	0.256908	0.503191	0.088*	
C8	−0.0906 (11)	0.4086 (4)	0.1504 (10)	0.084 (3)	
H8	−0.199147	0.405010	0.111488	0.101*	
C5	−0.2247 (11)	0.3018 (5)	0.0822 (10)	0.096 (3)	
H5	−0.290652	0.329367	0.048809	0.116*	
C12	0.4969 (13)	0.1855 (3)	0.3079 (12)	0.094 (3)	
H12	0.411937	0.167293	0.255928	0.113*	
C1	0.0190 (11)	0.2687 (4)	0.2006 (10)	0.080 (2)	
H1	0.123451	0.273124	0.249479	0.096*	
C14	0.7535 (13)	0.1862 (4)	0.4526 (11)	0.097 (3)	
H14	0.841828	0.168385	0.500949	0.117*	
C13	0.6271 (15)	0.1598 (4)	0.3807 (12)	0.096 (3)	
H13	0.628639	0.123990	0.380680	0.116*	
C4	−0.2814 (15)	0.2524 (6)	0.0566 (12)	0.110 (4)	
H4	−0.384491	0.247047	0.005943	0.132*	
C2	−0.0393 (14)	0.2198 (4)	0.1749 (12)	0.104 (3)	
H2	0.025503	0.191739	0.206548	0.125*	
C3	−0.1889 (15)	0.2122 (5)	0.1044 (12)	0.103 (4)	
H3	−0.228585	0.178916	0.088717	0.123*	
C10	−0.1408 (12)	0.5024 (4)	0.1353 (11)	0.115 (4)	
H10A	−0.184658	0.511753	0.210066	0.172*	
H10B	−0.226310	0.493619	0.061287	0.172*	
H10C	−0.081541	0.530940	0.111446	0.172*	
C21	0.369 (2)	0.5632 (6)	0.2438 (15)	0.164 (7)	
H21A	0.264632	0.558775	0.184657	0.196*	
H21B	0.364662	0.592990	0.299405	0.196*	
C22	0.492 (2)	0.5711 (7)	0.165 (2)	0.226 (11)	
H22A	0.454473	0.595840	0.095993	0.339*	
H22B	0.513178	0.539042	0.125400	0.339*	
H22C	0.589497	0.583392	0.221952	0.339*	

### Bis(benzoylacetonato)(ethanol)dioxidouranium(VI) Atomic displacement parameters (Å^2^)

**Table:**

	*U*^11^	*U*^22^	*U*^33^	*U*^12^	*U*^13^	*U*^23^
U1	0.0615 (2)	0.04146 (17)	0.0673 (2)	0.00267 (10)	−0.01179 (13)	−0.00574 (11)
O6	0.059 (3)	0.045 (3)	0.122 (5)	0.009 (2)	−0.018 (3)	−0.019 (3)
O7	0.111 (5)	0.051 (3)	0.085 (4)	0.005 (3)	−0.023 (3)	−0.006 (3)
O4	0.056 (3)	0.055 (3)	0.121 (5)	−0.006 (2)	−0.022 (3)	0.006 (3)
O2	0.143 (7)	0.072 (5)	0.064 (4)	0.009 (4)	−0.002 (4)	−0.002 (3)
O1	0.117 (6)	0.073 (4)	0.061 (4)	−0.010 (3)	0.009 (4)	−0.015 (3)
O3	0.075 (4)	0.068 (4)	0.122 (6)	0.016 (3)	−0.013 (4)	0.009 (4)
O5	0.074 (4)	0.056 (4)	0.166 (8)	0.002 (3)	−0.038 (4)	−0.030 (4)
C17	0.058 (4)	0.053 (4)	0.048 (4)	0.002 (3)	0.002 (3)	−0.001 (3)
C16	0.059 (4)	0.048 (4)	0.058 (4)	0.009 (3)	0.011 (3)	−0.001 (3)
C19	0.056 (5)	0.062 (5)	0.063 (5)	0.000 (4)	0.007 (4)	−0.014 (4)
C18	0.044 (4)	0.058 (5)	0.086 (6)	0.000 (3)	0.002 (4)	−0.007 (4)
C6	0.056 (5)	0.083 (6)	0.053 (4)	−0.004 (4)	0.009 (3)	−0.004 (4)
C7	0.058 (5)	0.061 (5)	0.063 (5)	0.001 (4)	−0.003 (4)	−0.002 (4)
C11	0.073 (5)	0.057 (5)	0.085 (6)	−0.008 (4)	0.007 (4)	−0.001 (4)
C9	0.082 (7)	0.080 (7)	0.077 (6)	0.017 (5)	−0.003 (5)	−0.003 (5)
C20	0.054 (5)	0.097 (7)	0.109 (8)	−0.025 (5)	0.013 (5)	−0.029 (6)
C15	0.068 (5)	0.066 (5)	0.086 (6)	0.017 (4)	0.013 (4)	0.005 (5)
C8	0.059 (5)	0.088 (7)	0.088 (7)	0.020 (5)	−0.019 (5)	−0.002 (6)
C5	0.078 (7)	0.114 (9)	0.090 (7)	−0.029 (6)	0.003 (5)	−0.003 (6)
C12	0.098 (7)	0.048 (5)	0.134 (9)	−0.016 (5)	0.022 (6)	−0.015 (6)
C1	0.069 (5)	0.074 (6)	0.093 (6)	−0.011 (5)	0.011 (5)	−0.010 (5)
C14	0.106 (8)	0.069 (7)	0.118 (9)	0.041 (6)	0.027 (7)	0.027 (6)
C13	0.134 (9)	0.047 (5)	0.119 (9)	0.012 (6)	0.050 (7)	0.012 (6)
C4	0.088 (8)	0.139 (11)	0.098 (8)	−0.050 (8)	0.009 (6)	−0.018 (8)
C2	0.107 (8)	0.078 (7)	0.132 (10)	−0.023 (6)	0.039 (7)	−0.011 (7)
C3	0.116 (9)	0.102 (9)	0.101 (9)	−0.061 (7)	0.049 (7)	−0.036 (7)
C10	0.102 (7)	0.086 (7)	0.132 (9)	0.051 (6)	−0.026 (7)	−0.011 (7)
C21	0.24 (2)	0.151 (14)	0.102 (11)	−0.078 (13)	0.038 (12)	−0.012 (10)
C22	0.154 (19)	0.30 (3)	0.23 (2)	−0.022 (15)	0.046 (17)	−0.070 (19)

### Bis(benzoylacetonato)(ethanol)dioxidouranium(VI) Geometric parameters (Å, º)

**Table:**

U1—O6	2.317 (5)	C20—H20B	0.9600
U1—O7	2.458 (5)	C20—H20C	0.9600
U1—O4	2.308 (5)	C15—H15	0.9300
U1—O2	1.779 (8)	C15—C14	1.374 (12)
U1—O1	1.759 (7)	C8—H8	0.9300
U1—O3	2.358 (7)	C5—H5	0.9300
U1—O5	2.369 (7)	C5—C4	1.376 (15)
O6—C17	1.279 (8)	C12—H12	0.9300
O7—H7	0.854 (10)	C12—C13	1.371 (14)
O7—C21	1.544 (15)	C1—H1	0.9300
O4—C7	1.246 (8)	C1—C2	1.371 (12)
O3—C9	1.253 (10)	C14—H14	0.9300
O5—C19	1.260 (10)	C14—C13	1.354 (14)
C17—C16	1.490 (10)	C13—H13	0.9300
C17—C18	1.389 (10)	C4—H4	0.9300
C16—C11	1.374 (11)	C4—C3	1.339 (16)
C16—C15	1.388 (10)	C2—H2	0.9300
C19—C18	1.373 (11)	C2—C3	1.338 (15)
C19—C20	1.484 (11)	C3—H3	0.9300
C18—H18	0.9300	C10—H10A	0.9600
C6—C7	1.481 (11)	C10—H10B	0.9600
C6—C5	1.388 (11)	C10—H10C	0.9600
C6—C1	1.366 (12)	C21—H21A	0.9700
C7—C8	1.414 (12)	C21—H21B	0.9700
C11—H11	0.9300	C21—C22	1.482 (9)
C11—C12	1.370 (12)	C22—H22A	0.9600
C9—C8	1.360 (14)	C22—H22B	0.9600
C9—C10	1.502 (12)	C22—H22C	0.9600
C20—H20A	0.9600		
			
O6—U1—O7	141.3 (2)	C19—C20—H20B	109.5
O6—U1—O3	146.3 (2)	C19—C20—H20C	109.5
O6—U1—O5	70.61 (19)	H20A—C20—H20B	109.5
O4—U1—O6	75.28 (18)	H20A—C20—H20C	109.5
O4—U1—O7	143.4 (2)	H20B—C20—H20C	109.5
O4—U1—O3	71.2 (2)	C16—C15—H15	120.1
O4—U1—O5	145.3 (2)	C14—C15—C16	119.8 (9)
O2—U1—O6	93.2 (3)	C14—C15—H15	120.1
O2—U1—O7	88.4 (3)	C7—C8—H8	117.6
O2—U1—O4	89.1 (3)	C9—C8—C7	124.8 (8)
O2—U1—O3	89.7 (3)	C9—C8—H8	117.6
O2—U1—O5	86.5 (4)	C6—C5—H5	119.3
O1—U1—O6	88.8 (3)	C4—C5—C6	121.4 (11)
O1—U1—O7	89.0 (2)	C4—C5—H5	119.3
O1—U1—O4	93.0 (3)	C11—C12—H12	120.2
O1—U1—O2	177.4 (3)	C11—C12—C13	119.7 (9)
O1—U1—O3	89.6 (3)	C13—C12—H12	120.2
O1—U1—O5	92.6 (3)	C6—C1—H1	118.9
O3—U1—O7	72.3 (2)	C6—C1—C2	122.2 (9)
O3—U1—O5	143.1 (2)	C2—C1—H1	118.9
O5—U1—O7	70.9 (2)	C15—C14—H14	119.7
C17—O6—U1	140.2 (5)	C13—C14—C15	120.5 (9)
U1—O7—H7	115.4 (16)	C13—C14—H14	119.7
C21—O7—U1	135.6 (7)	C12—C13—H13	119.8
C21—O7—H7	107.4 (17)	C14—C13—C12	120.3 (9)
C7—O4—U1	140.5 (6)	C14—C13—H13	119.8
C9—O3—U1	136.3 (6)	C5—C4—H4	119.9
C19—O5—U1	137.9 (5)	C3—C4—C5	120.3 (11)
O6—C17—C16	116.3 (6)	C3—C4—H4	119.9
O6—C17—C18	121.1 (7)	C1—C2—H2	119.8
C18—C17—C16	122.6 (7)	C3—C2—C1	120.3 (12)
C11—C16—C17	119.6 (7)	C3—C2—H2	119.8
C11—C16—C15	118.8 (7)	C4—C3—H3	120.0
C15—C16—C17	121.6 (7)	C2—C3—C4	120.0 (11)
O5—C19—C18	122.6 (8)	C2—C3—H3	120.0
O5—C19—C20	115.3 (8)	C9—C10—H10A	109.5
C18—C19—C20	122.0 (8)	C9—C10—H10B	109.5
C17—C18—H18	116.5	C9—C10—H10C	109.5
C19—C18—C17	126.9 (7)	H10A—C10—H10B	109.5
C19—C18—H18	116.5	H10A—C10—H10C	109.5
C5—C6—C7	124.4 (9)	H10B—C10—H10C	109.5
C1—C6—C7	119.7 (7)	O7—C21—H21A	109.9
C1—C6—C5	115.8 (9)	O7—C21—H21B	109.9
O4—C7—C6	116.0 (7)	H21A—C21—H21B	108.3
O4—C7—C8	121.8 (8)	C22—C21—O7	109.0 (17)
C8—C7—C6	122.3 (7)	C22—C21—H21A	109.9
C16—C11—H11	119.6	C22—C21—H21B	109.9
C12—C11—C16	120.8 (9)	C21—C22—H22A	109.5
C12—C11—H11	119.6	C21—C22—H22B	109.5
O3—C9—C8	125.3 (9)	C21—C22—H22C	109.5
O3—C9—C10	114.9 (9)	H22A—C22—H22B	109.5
C8—C9—C10	119.8 (9)	H22A—C22—H22C	109.5
C19—C20—H20A	109.5	H22B—C22—H22C	109.5
			
U1—O6—C17—C16	−179.6 (6)	C18—C17—C16—C15	13.4 (12)
U1—O6—C17—C18	0.3 (13)	C6—C7—C8—C9	−179.4 (9)
U1—O7—C21—C22	−90.4 (15)	C6—C5—C4—C3	0.4 (18)
U1—O4—C7—C6	177.7 (6)	C6—C1—C2—C3	0.2 (16)
U1—O4—C7—C8	−2.2 (16)	C7—C6—C5—C4	177.2 (9)
U1—O3—C9—C8	−5.2 (17)	C7—C6—C1—C2	−177.7 (9)
U1—O3—C9—C10	176.2 (7)	C11—C16—C15—C14	−0.9 (13)
U1—O5—C19—C18	−9.9 (17)	C11—C12—C13—C14	−2.4 (17)
U1—O5—C19—C20	172.1 (8)	C20—C19—C18—C17	178.2 (8)
O6—C17—C16—C11	12.1 (11)	C15—C16—C11—C12	−0.8 (13)
O6—C17—C16—C15	−166.8 (7)	C15—C14—C13—C12	0.7 (17)
O6—C17—C18—C19	4.2 (14)	C5—C6—C7—O4	−170.7 (8)
O4—C7—C8—C9	0.5 (17)	C5—C6—C7—C8	9.2 (14)
O3—C9—C8—C7	3.0 (19)	C5—C6—C1—C2	−1.2 (14)
O5—C19—C18—C17	0.3 (15)	C5—C4—C3—C2	−1.5 (19)
C17—C16—C11—C12	−179.7 (9)	C1—C6—C7—O4	5.5 (12)
C17—C16—C15—C14	178.0 (8)	C1—C6—C7—C8	−174.5 (9)
C16—C17—C18—C19	−176.0 (8)	C1—C6—C5—C4	0.9 (14)
C16—C11—C12—C13	2.5 (15)	C1—C2—C3—C4	1.2 (18)
C16—C15—C14—C13	1.0 (15)	C10—C9—C8—C7	−178.4 (10)
C18—C17—C16—C11	−167.8 (8)		

### Bis(benzoylacetonato)(ethanol)dioxidouranium(VI) Hydrogen-bond geometry (Å, º)

**Table:**

*D*—H···*A*	*D*—H	H···*A*	*D*···*A*	*D*—H···*A*
O7—H7···O2^i^	0.86 (6)	2.44 (5)	3.246 (10)	158 (3)

Symmetry code: (i) −*x*+1, −*y*+1, −*z*+1.

## References

[bb1] Ainscough, E. W., Brodie, A. M., Cresswell, R. J. & Waters, J. M. (1998). *Inorg. Chem.* **277**, 37–45.

[bb2] Al-Anber, M. A., Daoud, H. M., Rüffer, T. & Lang, H. (2011). *J. Mol. Struct.* **997**, 1–6.

[bb3] Almond, P. M. & Albrecht-Schmitt, T. E. (2003). *Inorg. Chem.* **42**, 5693–5698. 10.1021/ic034308d 12950219

[bb4] Arndt, S., Spaniol, T. P. & Okuda, J. (2002). *Chem. Commun.* pp. 896–897. 10.1039/b201613n 12123033

[bb5] Bean, A. C., Ruf, M. & Albrecht-Schmitt, T. E. (2001). *Inorg. Chem.* **40**, 3959–3963. 10.1021/ic010342l 11466054

[bb6] Belford, R. L., Chasteen, E. D., Hitchmbx, M. A., Ho’k, P. K., Pfluger, C. E. & Paul, I. C. (1969). *Inorg. Chem.* **8**, 1312–1319.

[bb7] Chakraborty, S., Dinda, S., Bhattacharyya, R. & Mukherjee, A. K. (2006). *Z. Kristallogr. Cryst. Mater.* **221**, 606–611.

[bb8] Chen, G. J., Chen, Ch. Q., Li, X. T., Ma, H. Ch. & Dong, Y. B. (2018). *Chem. Commun.* **54**, 11550–11553. 10.1039/c8cc07208f 30265269

[bb9] Cvrtila, I., Stilinović, V. & Kaitner, B. (2012). *Struct. Chem.* **23**, 587–594.

[bb10] Cvrtila, I., Stilinović, V. & Kaitner, B. (2013). *CrystEngComm*, **15**, 6585–6593.

[bb11] Dang, F. F., Lei, K. W., Wang, Y. W., Liu, W. Sh. & Sun, Y. X. (2006). *Anal. Sci. X-ray Struct. Anal. Online*, **22**, x279–x280.

[bb12] Döring, M., Ludwig, W., Uhlig, E., Wočadlo, S. & Müller, U. (1992). *Z. Anorg. Allg. Chem.* **611**, 61–67.

[bb13] Enriquez, A. E., Scott, B. L. & Neu, M. P. (2005). *Inorg. Chem.* **44**, 7403–7413. 10.1021/ic050578f 16212366

[bb14] Gatto, C. C., Lang, E. S., Jagst, A. & Abram, U. (2004). *Inorg. Chim. Acta*, **357**, 4349–4644.

[bb15] Groom, C. R., Bruno, I. J., Lightfoot, M. P. & Ward, S. C. (2016). *Acta Cryst.* B**72**, 171–179. 10.1107/S2052520616003954 PMC4822653 27048719

[bb16] Haider, S. Z., Malik, K. M. A., Rahman, A. & Hursthouse, M. B. (1983). *J. Bangladesh Acad. Sci.* **7**, 7–12.

[bb17] Hernandez, A., Chakraborty, I., Ortega, G. & Dares, C. J. (2022). *Acta Cryst.* E**78**, 40–43. 10.1107/S2056989021011063 PMC8739209 35079421

[bb18] Hon, P., Pfluger, C. E. & Belford, R. L. (1966). *Inorg. Chem.* **5**, 516–521.

[bb19] Hughes, K.-A. & Burns, P. C. (2003). *Acta Cryst.* C**59**, i7–i8. 10.1107/s0108270102021108 12506210

[bb20] Jung, Y. S., Lee, J. H., Song, K. & Kang, S. J. (1998). *Bull. Korean Chem. Soc.* **19**, 4484–4486.

[bb21] Kannan, S., Chetty, K. V., Venugopal, V. & Drew, G. B. (2004). *Dalton Trans. pp.* 3604–3610. 10.1039/b407824a 15510283

[bb22] Lennartson, A., Håkansson, M. & Jagner, S. (2007). *New J. Chem.* **31**, 344–347.

[bb23] McKinnon, J. J., Jayatilaka, D. & Spackman, M. A. (2007). *Chem. Commun. pp.* 3814–3816. 10.1039/b704980c 18217656

[bb24] Neu, M. P., Johnson, M. T., Matonic, J. H. & Scott, B. L. (2001). *Acta Cryst.* C**57**, 240–242. 10.1107/s0108270100017285 11250561

[bb25] Okeya, S., Asai, H., Ooi, S., Matsumoto, K., Kawaguchi, S. & Kuroya, H. (1976). *Inorg. Nucl. Chem. Lett.* **12**, 677–680.

[bb26] Oldham, W. J., Scott, B. L., Abney, K. D., Smith, W. H. & Costa, D. A. (2002). *Acta Cryst.* C**58**, m139–m140. 10.1107/s0108270101020492 11870280

[bb27] Perdih, F. (2014). *Struct. Chem.* **25**, 809–819.

[bb28] Pettinari, C., Marchetti, F., Pettinari, R., Gindulyte, A., Massa, L., Rossi, M. & Caruso, F. (2002). *Inorg. Chem.* pp. 1447–1455.

[bb29] Rigaku OD (2020). *CrysAlis PRO*. Rigaku Oxford Diffraction, Yarnton, England.

[bb30] Şahin, S. & Dege, N. (2021). *Polyhedron*, **205**, 115320–115330.

[bb31] Sheldrick, G. M. (2015*a*). *Acta Cryst.* A**71**, 3–8.

[bb32] Sheldrick, G. M. (2015*b*). *Acta Cryst.* C**71**, 3–8.

[bb33] Spackman, P. R., Turner, M. J., McKinnon, J. J., Wolff, S. K., Grimwood, D. J., Jayatilaka, D. & Spackman, M. A. (2021). *J. Appl. Cryst.* **54**, 1006–1011. 10.1107/S1600576721002910 PMC8202033 34188619

[bb34] Spek, A. L. (2020). *Acta Cryst.* E**76**, 1–11. 10.1107/S2056989019016244 PMC6944088 31921444

[bb35] Sykora, R. E. & Albrecht-Schmitt, T. E. (2003). *Inorg. Chem.* **42**, 2179–2181. 10.1021/ic026290x 12665344

[bb36] Takao, K. & Ikeda, Y. (2008). *Acta Cryst.* E**64**, m219–m220. 10.1107/S1600536807063799 PMC2915146 21200566

[bb37] Westrip, S. P. (2010). *J. Appl. Cryst.* **43**, 920–925.

[bb38] Xing, Y. H., Bai, F. Y., Aoki, K., Sun, Z. & Ge, M. F. (2007). *Inorg. Nano-Met. Chem.* **37**, 203–211.

[bb39] Yang, P. (2018*a*). *CSD Communication* (refcode HICRAP). CCDC, Cambridge, England.

[bb40] Yang, P. (2018*b*). *CSD Communication* (refcode HICRET). CCDC, Cambridge, England.

[bb41] Zou, F., Tang, X., Huang, Y., Wan, Sh., Lu, F., Chen, Z. N., Wu, A. & Zhang, H. (2016). *CrystEngComm*, **18**, 6624–6631.

